# Classifying Ruptured Middle Cerebral Artery Aneurysms With a Machine Learning Based, Radiomics-Morphological Model: A Multicentral Study

**DOI:** 10.3389/fnins.2021.721268

**Published:** 2021-08-11

**Authors:** Dongqin Zhu, Yongchun Chen, Kuikui Zheng, Chao Chen, Qiong Li, Jiafeng Zhou, Xiufen Jia, Nengzhi Xia, Hao Wang, Boli Lin, Yifei Ni, Peipei Pang, Yunjun Yang

**Affiliations:** ^1^Department of Radiology, The First Affiliated Hospital of Wenzhou Medical University, Wenzhou, China; ^2^Department of Radiology, Wenzhou Central Hospital, Wenzhou, China; ^3^The First School of Medicine, Wenzhou Medical University, Wenzhou, China; ^4^GE Healthcare China Co., Ltd., Shanghai, China; ^5^Department of Nuclear Medicine, The First Affiliated Hospital of Wenzhou Medical University, Wenzhou, China

**Keywords:** computed tomography angiography, decision support techniques, intracranial aneurysm, machine learning, middle cerebral artery, nomograms

## Abstract

**Objective:**

Radiomics and morphological features were associated with aneurysms rupture. However, the multicentral study of their predictive power for specific-located aneurysms rupture is rare. We aimed to determine robust radiomics features related to middle cerebral artery (MCA) aneurysms rupture and evaluate the additional value of combining morphological and radiomics features in the classification of ruptured MCA aneurysms.

**Methods:**

A total of 632 patients with 668 MCA aneurysms (423 ruptured aneurysms) from five hospitals were included. Radiomics and morphological features of aneurysms were extracted on computed tomography angiography images. The model was developed using a training dataset (407 patients) and validated with the internal (152 patients) and external validation (73 patients) datasets. The support vector machine method was applied for model construction. Optimal radiomics, morphological, and clinical features were used to develop the radiomics model (R-model), morphological model (M-model), radiomics-morphological model (RM-model), clinical-morphological model (CM-model), and clinical-radiomics-morphological model (CRM-model), respectively. A comprehensive nomogram integrating clinical, morphological, and radiomics predictors was generated.

**Results:**

We found seven radiomics features and four morphological predictors of MCA aneurysms rupture. The R-model obtained an area under the receiver operating curve (AUC) of 0.822 (95% CI, 0.776, 0.867), 0.817 (95% CI, 0.744, 0.890), and 0.691 (95% CI, 0.567, 0.816) in the training, temporal validation, and external validation datasets, respectively. The RM-model showed an AUC of 0.848 (95% CI, 0.810, 0.885), 0.865 (95% CI, 0.807, 0.924), and 0.721 (95% CI, 0.601, 0.841) in the three datasets. The CRM-model obtained an AUC of 0.856 (95% CI, 0.820, 0.892), 0.882 (95% CI, 0.828, 0.936), and 0.738 (95% CI, 0.618, 0.857) in the three datasets. The CRM-model and RM-model outperformed the CM-model and M-model in the internal datasets (*p* < 0.05), respectively. But these differences were not statistically significant in the external dataset. Decision curve analysis indicated that the CRM-model obtained the highest net benefit for most of the threshold probabilities.

**Conclusion:**

Robust radiomics features were determined related to MCA aneurysm rupture. The RM-model exhibited good ability in classifying ruptured MCA aneurysms. Integrating radiomics features into conventional models might provide additional value in ruptured MCA aneurysms classification.

## Introduction

Middle cerebral artery (MCA) aneurysm is the most common subtype of unruptured aneurysms (Huttunen et al., 2010; Can et al., 2015). With the improvement of imaging techniques, unruptured aneurysms have become more frequently detected (Greving et al., 2014). But therapeutic decision-making for them is controversial. On the one hand, many unruptured aneurysms stay asymptomatic and never rupture (Korja et al., 2014). The prophylactic treatment such as current endovascular and microsurgical interventions carries the risk of procedure-related complications (Naggara et al., 2010; Zhu et al., 2020). On the other hand, once the aneurysm ruptures, the outcome is catastrophic (Vlak et al., 2011). Therefore, it is vital to screen out rupture-prone aneurysms.

Previous studies have identified that morphological features were associated with aneurysms rupture (Lindgren et al., 2016; Zhu et al., 2020). Researchers have constructed various computational methods using morphological features to evaluate the aneurysms-rupture risk (Zhang et al., 2019; Tanioka et al., 2020; Zhu et al., 2020). However, those morphological features are measured on two-dimensional images and might be affected by different readers or projections. It could impair the comparability of results.

Radiomics is an emerging technology that extracts high-throughput data from medical images (Zhou et al., 2018; Hua et al., 2020; Tomaszewski and Gillies, 2021). Recently, radiomics is frequently used in cerebrovascular disease researches (Chen et al., 2021; Zhu D. et al., 2021; Zhu D. Q. et al., 2021). Several researchers have scoped to the whole-brain aneurysms and proved that radiomics features were related to aneurysms rupture status (Liu et al., 2019; Ou et al., 2021). Regretfully, they did not analyze the features’ robustness, which can be easily affected by a slight change in image-scanning protocols or regions of interest (ROIs) segmentation (Mackin et al., 2015; Choe et al., 2019). Moreover, the predictive ability of radiomics in those studies was not validated by any external validation dataset, which leads to the uncertainness of their results’ generalizability (Collins et al., 2015; Lambin et al., 2017).

To the best of our knowledge, few studies have predicted the rupture of the location-specific aneurysm with robust radiomics features. In this study, we included a large sample of 668 MCA aneurysms. We aimed to (1) determine whether there are robust radiomics features that can classify ruptured MCA aneurysms; and (2) evaluate the additional value of combining morphological and radiomics features in classifying ruptured MCA aneurysm.

## Materials and Methods

Our study was approved by the Medical Ethics Committee of our hospital.

### Study Population and Clinical Data

We performed a retrospective and multicentral study using the data from five hospitals (hospitals A, B, C, D, and E). MCA aneurysms with available computed tomography angiography (CTA) data were included. Exclusion criteria were as follows: fusiform MCA aneurysms, aneurysms combined with vascular diseases (such as Moyamoya disease and arteriovenous malformations), aneurysms with a size <3 mm, aneurysms with poor-quality images and patients underwent surgery or interventional therapy before CTA examination (see Supplementary Methods, Supplementary Digital Content 1, which illustrates details about CTA image scanning).

Patients with MCA aneurysms seen in hospital A from January 2009 to December 2019 were allocated to the training and the internal validation datasets. The training dataset encompassed the patients from the earlier period (2009–2017). The patients from the more recent period (2018–2019) were attributed to the temporal validation dataset (internal validation dataset) (Collins et al., 2015; Al-Shahi Salman et al., 2018). For external validation, MCA aneurysms cases in four hospitals, including hospital B (from January 2018 to December 2020), hospital C (from January 2018 to December 2020), hospital D (from January 2017 to October 2019), and hospital E (from September 2019 to March 2020), were merged to one external validation dataset (Lambin et al., 2017).

Clinical data such as age, sex, history of hypertension (a diagnosis of hypertension previously made by another physician or use of antihypertensive drugs), cigarette smoking (previous smoker or current smoker), and aneurysm side were collected. Rupture status of aneurysms was evaluated using the following criterion: (1) for patients with subarachnoid hemorrhage (SAH), aneurysms adjacent to the cisternal clots were judged ruptured, and those aneurysms not adjacent to the cisternal clots were judged on digital subtraction angiography (DSA). (2) Asymptomatic patients without SAH were identified to be unruptured (Shi et al., 2021).

### Morphological Predictors Discovery

**FIGURE 1:**
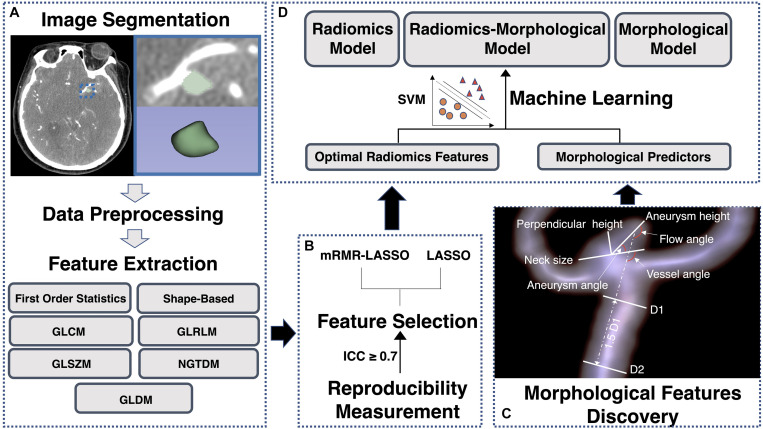
A flowchart of radiomics analysis and radiomics-clinical model construction. **(A)** The process of regions of interest (ROIs) segmentation; **(B)** the process of optimal radiomics features detection; **(C)** the process of morphological predictors discovery; and **(D)** the process of machine learning models development and validation. GLCM, gray level co-occurrence matrix; GLDM, gray level dependence matrix; GLRLM, grey level run-length matrix; GLSZM, gray level size zone matrix; NGTDM, neighboring gray tone difference matrix; SVM, support vector machine.

Morphological features such as aneurysm location (divided into M1, the proximal segment of the middle cerebral artery; Mbif, main middle cerebral artery bifurcation; Mdist, distal middle cerebral artery), aneurysm size, vessel size, aneurysm height, perpendicular height, aspect ratio (AR), size ratio (SR), aneurysm angle, flow angle, vessel angle, daughter dome, and irregular shape were measured as described in previous studies (Can et al., 2015; Chen et al., 2020; Zhu et al., 2020; Figure 1C; see Supplementary Methods, Supplementary Digital Content 2, which illustrates detailed definitions of morphological features).

We implemented univariate analysis to find morphological factors that were associated with MCA aneurysm rupture. After that, the multivariable logistic regression was performed to identify independent morphological predictors of MCA aneurysm rupture.

### Optimal Radiomics Signature Detection

The workflow process of radiomics analysis is shown in Figures 1A,B. ROIs of aneurysms were manually segmented by a neuroradiologist on each slice of CTA images (Figure 1A). Then, 50 aneurysms were randomly selected to be re-segmented by another neuroradiologist. We calculated the inter-class correlation coefficient (ICC) to evaluate the inter-observer reproducibility.

As the images were acquired from different CT scanners with different parameters, we performed data preprocessing before radiomics feature extraction (Lambin et al., 2017; Morin et al., 2018; Chen et al., 2021). Image resampling and gray-level discretization were used to reduce the variability of radiomics features (Shafiq-Ul-Hassan et al., 2018). A total of 1316 radiomics features were extracted from each ROI (see Supplementary Digital Content 3, which illustrates the possible pathophysiologic meaning of the features). All radiomics features were standardized by *z*-score to eliminate unit limits of each feature (Yang et al., 2019; see Supplementary Methods, Supplementary Digital Content 4, for further details about radiomics analysis).

Figure 1B indicates the feature selection procedure. Firstly, features with poor reproducibility (an ICC of <0.7) were excluded. After that, we used two different kinds of schemes to select informative radiomics features: (1) the minimum redundancy maximum relevance (mRMR) method was performed to rank the top 50 rupture-associated features while minimizing intra-feature correlation (Ding and Peng, 2005; Castiglioni et al., 2019), and then we used the least absolute shrinkage and selection operator (LASSO) method to select optimal features from those 50 features (Sauerbrei et al., 2007) (“mRMR-LASSO method”); (2) only LASSO method was used to selected optimal features (“LASSO method”) (see Supplementary Methods, Supplementary Digital Content 5, for further detailed information of mRMR and LASSO). We applied the logistics regression model to build radiomics signatures. Discrimination ability of the “mRMR-LASSO model” and “LASSO model” were compared. The features with better performance were used for subsequent analysis. Moreover, we calculated the Rad score through a linear combination of selected features by multiplying with their LASSO coefficients (Huang et al., 2016).

### Machine Learning Models Development and Validation

Support vector machine (SVM) is a supervised machine learning method that classifies data points by maximizing the distance between classes in a high-dimensional space (Orru et al., 2012). We applied SVM with a 10-fold cross-validation to construct models. As shown in Figure 1D, optimal radiomics features were introduced into the radiomics model (R-model). The morphological predictors of aneurysm rupture were introduced into the morphological model (M-model). Optimal radiomics features and morphological features were put together to generate the radiomics-morphological model (RM-model). Models were trained using the training dataset and validated in the temporal and external validation datasets. Variance inflation factor (VIF) was used to detect multicollinearity of the enrolled features, and a VIF of ≥5 was considered as multicollinearity (Akinwande et al., 2015).

The model performance was evaluated using the receiver operating characteristic (ROC) curve. The DeLong test was used for comparisons of an area under the receiver operating curves (AUCs) of different models (DeLong et al., 1988). The decision curve analysis (DCA) was applied to assess the clinical utility of the models. Besides, net reclassification improvement (NRI) (Pencina et al., 2011) was calculated to evaluate the improvement in the discrimination ability of different models.

### Nomogram Construction and Evaluation

To provide an easy and visualized rupture risk-scoring system, we constructed a comprehensive nomogram. The top five rupture-associated factors among the Rad score, clinical and morphological features were selected using the mRMR method. These five factors were used to generate the nomogram. The discrimination of the nomogram was assessed with ROC curves. The agreement between predicted rupture and observed rupture was evaluated using the calibration curve and the Hosmer–Lemeshow test (Kramer and Zimmerman, 2007). The discrimination and calibration of the nomogram were appraised in the training and validation datasets.

### Statistical Analysis

Categorical variables are presented as counts (with percentages), while continuous variables are presented as medians [interquartile range, (IQR)]. We used Student *t*-tests or Mann–Whitney U tests to evaluate the differences in continuous variables. Differences in categorical variables were assessed using the χ^2^ test or Fisher exact test (two-tailed). A *p*-value of <0.05 indicates a statistical difference. Statistical analysis and model construction were conducted using SPSS (version 24.0) and R (version 3.6.1).

## Results

### Clinical and Morphological Characteristics

A total of 632 patients with 668 MCA aneurysms (423 ruptured aneurysms) from five hospitals were included in our study. Multiple aneurysms were presented in 132 (20.9%) patients. Thirty-two of the 132 (24.2%) patients have bilateral MCA aneurysms. There were 407, 152, and 73 patients with 438, 155, and 75 aneurysms in the training, temporal validation, and external validation datasets. Table 1 shows the clinical and morphological characteristics of the training dataset (see Supplementary Digital Content 6, which shows characteristics of validation datasets). In univariate analysis, patients with ruptured aneurysms were younger and were less common to have a history of hypertension (*p* < 0.05).

**TABLE 1:** Baseline characteristics of patients in the training dataset.

Variables	Unruptured (*N* = 141)	Ruptured (*N* = 297)	*p*-Value
Age^*a*^	59.0 (53.0, 69.0)	55.0 (48.0, 64.3)	0.004
Female^*a*^	77.0 (54.6%)	173.0 (58.2%)	0.472
Hypertension^*b*^	75.0 (68.8%)	149.0 (55.4%)	0.016
Smoking^*c*^	30.0 (28.0%)	77.0 (28.6%)	0.909
Location			0.016
M1	51.0 (36.2%)	72.0 (24.2%)	
Mbif	85.0 (60.3%)	219.0 (73.7%)	
Mdist	5.0 (3.5%)	6.0 (2.0%)	
Side			0.862
Right	81.0 (57.4%)	168.0 (56.6%)	
Left	60.0 (42.6%)	129.0 (43.4%)	
Multiplicity	63.0 (44.7%)	66.6 (22.2%)	<0.001
Vessel size (mm)	2.5 (2.1, 2.8)	2.4 (2.0, 2.6)	0.002
Aneurysm size (mm)	5.6 (4.0, 7.8)	6.7 (5.0, 9.1)	0.001
Neck size (mm)	4.2 (3.3, 5.5)	3.9 (3.1, 4.8)	0.018
Aspect ratio	0.8 (0.5, 1.1)	1.0 (0.8, 1.4)	<0.001
Size ratio	1.6 (1.0, 2.3)	2.3 (1.6, 3.4)	<0.001
Aneurysm height (mm)	4.1 (2.6, 5.4)	5.1 (3.9, 6.9)	<0.001
Perpendicular height (mm)	3.3 (2.3, 4.6)	4.1 (3.0, 5.5)	<0.001
Aneurysm angle (°)	65.4 (53.5, 81.4)	61.4 (48.1, 76.5)	0.014
Vessel angle (°)	57.1 (37.4, 77.7)	64.3 (41.4, 78.4)	0.134
Flow angle (°)	135.8 (111.4, 158.5)	137.8 (116.2, 159.2)	0.319
Daughter dome	18.0 (12.8%)	102.0 (34.3%)	<0.001
Irregular shape	48.0 (34.0%)	179.0 (60.3%)	<0.001

*^a^3/438 (0.68%) missing values. ^b^60/438 (13.70%) missing values. ^c^62/438 (14.16%) missing values. M1, the proximal segment of the middle cerebral artery; Mbif, main middle cerebral artery bifurcation; Mdist, distal middle cerebral artery.*

For morphological features, the mean ICC value of the nine morphological features was 0.924. The kappa value of the irregular shape and daughter dome is 0.603 and 0.838, respectively (*p*-value for all <0.001). Vessel size, aneurysm size, neck size, AR, SR, aneurysm height, perpendicular height, aneurysm angle, irregular shape, and daughter dome were associated with aneurysm rupture (*p* < 0.05). The result of multivariate analysis (Table 2) indicates that SR [odds ratio (OR), 1.607 (95% CI, 1.309, 1.973); *p* < 0.001], neck size [OR, 0.690 (95% CI, 0.596, 0.799), *p* < 0.001], multiplicity [OR, 0.389 (95% CI, 0.244, 0.621), *p* < 0.001], and daughter dome [OR, 2.987 (95% CI, 1.650, 5.406), *p* < 0.001] were independent predictors of MCA aneurysms rupture.

**TABLE 2:** Univariate and multivariable analysis of morphological and clinical features associated with aneurysm rupture.

Variables	Univariate analysis			Multivariate analysis		
	Odds ratio	95% CI	*p*-Value	Odds ratio	95% CI	*p*-Value
Neck size (mm)	0.864	0.770, 0.970	0.018	0.690	0.596, 0.799	<0.001
Daughter dome	3.574	2.063, 6.192	<0.001	2.987	1.650, 5.406	<0.001
Size ratio	1.478	1.240, 1.761	<0.001	1.607	1.309, 1.973	<0.001
Multiplicity	0.354	0.230, 0.544	<0.001	0.389	0.244, 0.621	<0.001
Aneurysm height (mm)	1.169	1.074, 1.272	<0.001	–	–	0.407
Location	1.825	1.178, 2.827	0.016	–	–	0.385
Aneurysm size (mm)	1.080	1.009, 1.156	0.001	–	–	0.735
Aspect ratio	2.813	1.754, 4.511	<0.001	–	–	0.814
Vessel size (mm)	0.526	0.357, 0.776	0.002	–	–	0.747
Perpendicular height (mm)	1.110	1.015, 1.212	<0.001	–	–	0.731
Aneurysm angle (°)	0.987	0.976, 0.998	0.014	–	–	0.215
Irregular shape	2.939	1.934, 4.468	<0.001	–	–	0.100
Hypertension	0.625	0.387, 1.011	0.016	–	–	0.055
Age	0.974	0.954, 0.994	0.004	–	–	0.012

*CI, confidence interval.*

### Optimal Radiomics Features Detection

The mean ICC value of the overall 1316 radiomics features was 0.751. Eight hundred and eighty-one radiomics features showed a high interobserver agreement (an ICC value of ≥0.7). As shown in Table 3, the “mRMR-LASSO model” presented an AUC of 0.767 and 0.828 in the training and temporal validation dataset, respectively. The “mRMR-LASSO model” presented a higher discrimination ability than the “LASSO model.” Therefore, those features selected using the “mRMR-LASSO method” were used for R-model construction. The Rad score was calculated (see Supplementary Results, Supplementary Digital Content 7, which indicates the Rad score calculation formula).

**TABLE 3:** Performance of the “LASSO model” and “mRMR-LASSO model.”

Datasets	Method	Feature count	AUC (95% CI)	ACC	SEN	SPE	*p*-Value
Training dataset	LASSO	7	0.693 (0.638, 0.747)	0.717	0.811	0.518	0.003
	mRMR-LASSO	7	0.767 (0.718, 0.816)	0.774	0.869	0.574	
Temporal validation dataset	LASSO	7	0.767 (0.689, 0.845)	0.735	0.735	0.736	0.092
	mRMR-LASSO	7	0.828 (0.759, 0.897)	0.806	0.928	0.667	

*p-Values were derived from the DeLong test comparing AUCs between radiomics signatures built by two feature selection methods. AUC, area under the receiver operating curve; ACC, accuracy; CI, confidence interval; SEN, sensitivity; SPE, specificity.*

### Machine Learning Models Construction and Evaluation

**FIGURE 2:**
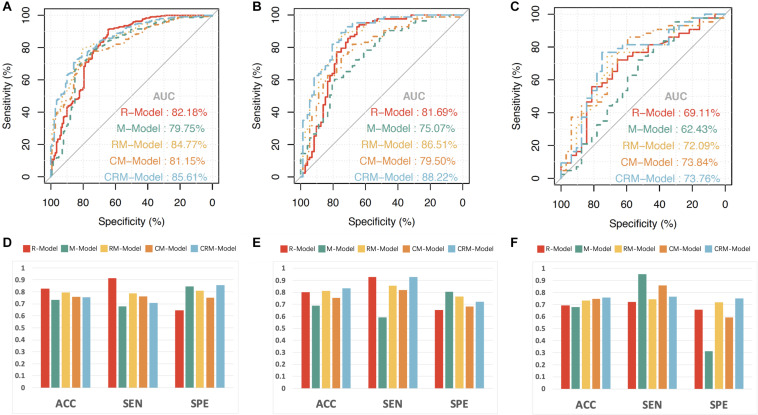
The receiver operating characteristic (ROC) curves, accuracy, sensitivity, and specificity of the radiomics model (R-model), morphological model (M-model), radiomics-morphological model (RM-model), clinical-morphological model (CM-model); and clinical-radiomics-morphological model (CRM-model). The ROC curves of the three models in training **(A)**, temporal validation **(B)**, and external validation datasets **(C)**. The accuracy, sensitivity, and specificity of the three models in the training **(D)**, temporal validation **(E)**, and external validation datasets **(F)**. ACC, accuracy; SEN, sensitivity; SPE, specificity.

The performance of the models was shown in Table 4 and Figure 2. The R-model obtained an AUC of 0.822 (95% CI, 0.776, 0.867) in the training dataset. In the temporal and external validation dataset, the R-model presented an AUC of 0.817 (95% CI, 0.744, 0.890) and 0.691 (95% CI, 0.567, 0.816), respectively. Four morphological predictors, including SR, neck size, multiplicity, and daughter dome, were used to construct the M-model. The M-model obtained an AUC of 0.798 (95% CI, 0.749, 0.846), 0.751 (95% CI, 0.674, 0.828), and 0.624 (95% CI, 0.490, 0.759) in the training, temporal, and external validation datasets, respectively. The *p*-values from the DeLong test of the statistical comparison of the ROC curves are given in Table 5.

**TABLE 4:** Performance of the radiomics, morphological, radiomics-morphological, clinical-morphological, and clinical-radiomics-morphological models.

Datasets	Models	AUC (95% CI)	ACC	SEN	SPE	PPV	NPV
Training dataset	R-model	0.822 (0.776, 0.867)	0.826	0.912	0.645	0.844	0.778
	M-model	0.798 (0.749, 0.846)	0.733	0.680	0.844	0.902	0.556
	RM-model	0.848 (0.810, 0.885)	0.795	0.788	0.809	0.897	0.644
	CM-model	0.811 (0.770, 0.853)	0.758	0.761	0.752	0.866	0.599
	CRM-model	0.856 (0.820, 0.892)	0.756	0.707	0.858	0.913	0.582
Temporal validation dataset	R-model	0.817 (0.744, 0.890)	0.800	0.928	0.653	0.755	0.887
	M-model	0.751 (0.674, 0.828)	0.690	0.590	0.806	0.778	0.630
	RM-model	0.865 (0.807, 0.924)	0.813	0.855	0.764	0.807	0.821
	CM-model	0.795 (0.723, 0.867)	0.755	0.819	0.681	0.747	0.766
	CRM-model	0.882 (0.828, 0.936)	0.832	0.928	0.722	0.794	0.897
External validation dataset	R-model	0.691 (0.567, 0.816)	0.693	0.721	0.656	0.738	0.636
	M-model	0.624 (0.490, 0.759)	0.680	0.953	0.313	0.651	0.833
	RM-model	0.721 (0.601, 0.841)	0.733	0.744	0.719	0.780	0.676
	CM-model	0.738 (0.621, 0.855)	0.747	0.860	0.594	0.740	0.760
	CRM-model	0.738 (0.618, 0.857)	0.760	0.767	0.750	0.805	0.706

*R-model, radiomics model; M-model, morphological model; RM-model, radiomics-morphological model; CM-model, clinical-morphological model; CRM-model, clinical-radiomics-morphological model; AUC, area under the receiver operating curve; ACC, accuracy; CI, confidence interval; PPV, positive predictive value; NPV, negative predictive value. SEN, sensitivity; SPE, specificity.*

**TABLE 5:** The *p*-values of the DeLong test of the statistical comparison of the ROC curves in all datasets.

	Training dataset	Temporal validation dataset	External validation dataset
R-model vs. M-model	0.458	0.211	0.457
M-model vs. RM-model	0.041	0.005	0.224
R-model vs. RM-model	0.176	0.115	0.559
R-model vs. CM-model	0.743	0.685	0.603
M-model vs. CM-model	0.515	0.212	0.106
R-model vs. CRM-model	0.078	0.041	0.407
M-model vs. CRM-model	0.018	0.002	0.152
RM-model vs. CRM-model	0.176	0.096	0.378
CM-model vs. CRM-model	0.038	0.018	0.993

*R-model, radiomics model; M-model, morphological model; RM-model, radiomics-morphological model; CM-model, clinical-morphological model; CRM-model, clinical-radiomics-morphological model.*

The optimal radiomics features and morphological predictors were enrolled in the RM-model. The RM-model exhibited good ability in classifying ruptured MCA aneurysms, with an AUC of 0.848 (95% CI, 0.810, 0.885), accuracy of 0.795, sensitivity of 0.788, and specificity of 0.809 in the training dataset. We further validated the RM-model in two validation datasets. The AUC of RM-model for ruptured MCA aneurysms classification was 0.865 (95% CI, 0.807, 0.924) and 0.721 (95% CI, 0.601, 0.841) in the temporal and external validation datasets, respectively (Figure 2 and Table 4). Multicollinearity was not observed between those selected radiomics features and morphological predictors (VIF for all <2).

Compared with the single R-model and M-model, the RM-model achieved a higher AUC. In the training dataset, the RM-model outperformed the M-model [AUC (95% CI), 0.848 (0.810, 0.885) vs. 0.798 (0.749, 0.846), *p* = 0.041]. In the validation datasets, the RM-model tended to have a better presented higher AUC than the single M-model. The difference was statistically significant in the temporal validation dataset (*p* < 0.005) while it was not statistically significant in the external validation dataset (*p* = 0.224).

We further added clinical features (hypertension, smoking, age, and sex) into the M-model and RM-model to construct the clinical-morphological model (CM-model) and clinical-radiomics-morphological model (CRM-model). As it was shown in Table 4 and Figure 2, the CRM-model obtained an AUC (95% CI) of 0.856 (0.820, 0.892), 0.882 (0.828, 0.936), and 0.738 (0.618, 0.857) in the three datasets, respectively. The CRM-model outperformed the CM-model in the training and temporal validation datasets (*p* < 0.05). However, the difference was not observed in the external validation dataset. We further calculated NRI to evaluate the improvement of discrimination by adding radiomics features to CM-model. We found that adding radiomics features to CM-mode improved the net reclassification indices in the three datasets (additive NRI, 52.40%, 89.66%, and 24.62%, respectively). This indicated that compared to the CM-model, the CRM-model correctly reclassified 52.40%, 89.66%, and 24.62% cases in the three datasets, respectively.

**FIGURE 3:**
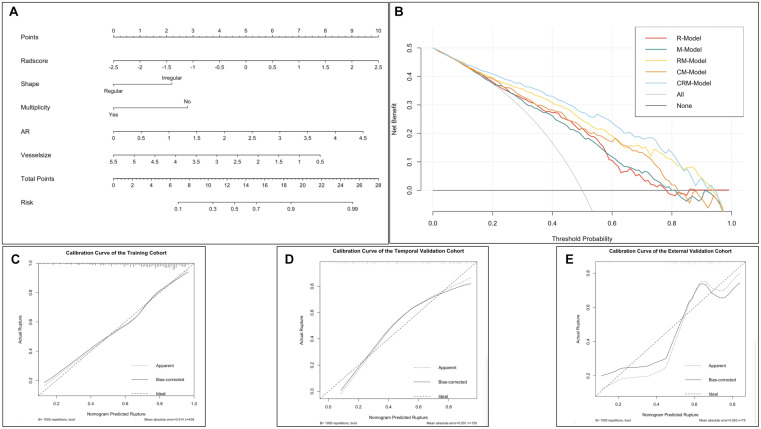
The comprehensive nomogram for classifying ruptured MCA aneurysm and decision curve analysis in the overall patients. **(A)** The comprehensive nomogram for predicting aneurysm rupture. **(B)** Decision curve analysis in overall patients. The *y*-axis indicates the net benefit; the *x*-axis indicates threshold probability. The gray line represents the assumption that all aneurysms rupture. The black line represents the assumption that no aneurysm ruptures. The red line, green line, gold line, orange line, and blue line represent the net benefit of the radiomics model (R-model), morphological model (M-model), radiomics-morphological model (RM-model), clinical-morphological model (CM-model), and clinical-radiomics-morphological model (CRM-model), respectively. Calibration of the nomogram in the training **(C)**, temporal validation **(D)**, and external validation datasets **(E)**.

With respect to clinical utility, the DCA (Figure 3B) indicated that the RM-model had a higher overall net benefit in distinguishing ruptured aneurysms than the single R-model and M-model for most of the threshold probabilities. The CRM-model obtained the highest net benefit for most of the threshold probabilities. Combining radiomics features to conventional models resulted in an extra net-benefit compared with the M-model and CM-model.

### Nomogram Construction and Evaluation

The shape, vessel size, AR, multiplicity, and Rad score were incorporated into the comprehensive nomogram (Figure 3A). The nomogram presented satisfying discrimination ability with an AUC of 0.771 (95% CI, 0.723, 0.818), 0.823, (95% CI, 0.757, 0.889), and 0.709 (95% CI, 0.592, 0.827) in the training, temporal validation, and external validation datasets, respectively. The calibration curve (Figures 3C–E) and the Hosmer–Lemeshow test (*p* = 0.731, 0.325, and 0.067, in the three datasets, respectively) indicate good calibration.

## Discussion

This study developed and validated ruptured MCA aneurysms classification models based on radiomics and morphological features in a large and multicentral dataset (a total of 668 MCA aneurysms were enrolled). It was proved that robust radiomics features extracted from CTA images could classify ruptured MCA aneurysms. The RM-model could identify more than 74% ruptured MCA aneurysms with a specificity of 72–81%. Additionally, we provided an easy and visualized rupture risk-scoring system for MCA aneurysm patients through the nomogram.

In this study, a total of 1316 radiomics features were extracted for each aneurysm. Among those numerous radiomics features, the features indicating image heterogeneity (e.g., Dependence Non-Uniformity Normalized, Cluster Shade, and Variance) were screened out as the optimal signature for ruptured aneurysms classification. This is in agreement with the study of Ou et al. (2021). The possible explanation for this is that CTA image heterogeneity is caused by the contrast maldistribution in the vessel lumen. The uneven distribution of contrast indicates turbulent flow (George et al., 2016; Aghayev et al., 2018; Ou et al., 2021), which was one of the risk factors for aneurysms rupture (Lv et al., 2020). Therefore, we speculate that CTA-derived radiomics features might imply the hemodynamics condition of aneurysms.

Unlike former research (Liu et al., 2019; Ou et al., 2021), shape radiomics features were not selected for model construction. We exclude some shape features before the feature selection due to their poor interobserver agreement (e.g., the shape radiomics features Sphericity and Flatness obtained an ICC value of 0.536 and 0.590, respectively). Only highly stable features were used in the model construction procedure. The temporal and external validation datasets further verified the robustness and generalizability of the results.

It is generally acknowledged that vessel wall degradation and abnormal morphological condition are related to aneurysms’ rupture. We found that daughter dome, multiplicity, neck size, and SR were independent predictors for MCA aneurysm rupture, which have been reported by other researchers (Can et al., 2015; Zhang et al., 2019; Ou et al., 2020; Tanioka et al., 2020; Zhu et al., 2020).

Both radiomics and morphological features originate from CTA images, but their biological meanings and analysis procedures are different. Radiomics features describe the shape and texture characteristics of aneurysms from the micro point of view (Lambin et al., 2017; Xu et al., 2019), while the morphological features measure the macroscopic observation of the aneurysms. Radiomics features are three-dimensional derived, high-through biomarkers but lacking clinical interpretability. Meanwhile, those morphological features are two-dimensional measured features with relatively less information, but they are still fundamental tools for aneurysm evaluation. Though the statistically significant improvement in AUC of adding radiomics was not observed in the external dataset, considering its objectivity and its extra net-benefit in DCA, radiomics could be a possible choice in clinical practice, enabling better patient management.

Aneurysms rupture leads to catastrophic consequences. Preventive treatment for high rupture risk patients is necessary. However, preventive treatments for low rupture risk patients may cause more harm than good due to operation-related complications (Naggara et al., 2010). Therefore, except for stratifying rupture-prone aneurysms, accurate identification of aneurysms at a relatively low rupture risk is also essential. Compared to the CM-model (which is routinely used in clinical practice), the CRM-model obtained a higher specificity, positive predictive value (PPV), and the CRM-model correctly reclassified 52.40%, 89.66%, and 24.62% patients in the three datasets, respectively. This indicated that adding radiomics features to conventional models might not only classify ruptured aneurysms but also help in recognizing unruptured aneurysms, which may reduce unnecessary treatment for unruptured aneurysms patients.

There are some limitations in this study. First, this is a retrospective, cross-sectional study without the longitudinal follow-up of aneurysms, which might inherently cause biases. Second, the aneurysms’ morphological changes after rupture were not considered because it is hard to collect the morphological change before and after the aneurysm rupture due to ethical issues. Third, most patients with SAH history were excluded because of their surgery experience. This could cause potential selection bias. Forth, images from external validation datasets contain different scanning protocols, which might affect the validation results. We performed image resampling and gray-level discretization to reduce the variability of images. Moreover, samples from the other four hospitals were merged into one external validation dataset. The good results further indicated the robustness of the models.

## Conclusion

In conclusion, we analyzed the MCA aneurysms rupture by clinical, radiomics, and morphological features using multicentral data. We answered two critical questions: (1) Robust radiomics features could classify ruptured MCA aneurysms. (2) The integration of radiomics into conventional clinical and morphological models might provide additional benefit in ruptured MCA aneurysms classification. An easy and visualized rupture risk-scoring nomogram was generated. This may aid in the rupture-risk assessment of MCA aneurysms.

## Data Availability Statement

The original contributions presented in the study are included in the article/Supplementary Material, further inquiries can be directed to the corresponding author/s.

## Ethics Statement

The studies involving human participants were reviewed and approved by the Medical Ethics Committee of the First Affiliated Hospital of Wenzhou Medical University. Written informed consent for participation was not required for this study in accordance with the national legislation and the institutional requirements.

## Author Contributions

DZ wrote the manuscript. DZ, PP, and YC interpreted the data and prepared the tables and figures. JZ, YC, and KZ revised the manuscript for intellectual content. CC, QL, JZ, NX, HW, BL, YN, and XJ acquired the data. YY contributed to the conception and the design of the study. All co-authors read and revised the manuscript.

## Conflict of Interest

PP was employed by the company GE Healthcare China Co., Ltd., Shanghai. The remaining authors declare that the research was conducted in the absence of any commercial or financial relationships that could be construed as a potential conflict of interest.

## Publisher’s Note

All claims expressed in this article are solely those of the authors and do not necessarily represent those of their affiliated organizations, or those of the publisher, the editors and the reviewers. Any product that may be evaluated in this article, or claim that may be made by its manufacturer, is not guaranteed or endorsed by the publisher.
