# Supplementary material for: Classifying Ruptured Middle Cerebral Artery Aneurysms With a Machine Learning Based, Radiomics-Morphological Model: A Multicentral Study
Source: Front Neurosci. 2021 Aug 11;15:721268. doi: 10.3389/fnins.2021.721268 (PMC8385786; doi:10.3389/fnins.2021.721268)
Supplement: Supplementary file 1 [file Data_Sheet_1.PDF]

Supplemental Digital Content

Supplemental Digital Content 1: Details about CTA image scanning external data information

1. Hospital A (from January 2009 to December 2019): All CTA images were acquired using a 64-channel multidetector CT scanner (LightSpeed VCT 64; GE Medical Systems, Milwaukee, WI, USA) with scan thickness of 0.625 mm, reconstruction interval of 0.625 mm, tube voltage of 100 kV, tube current of 500 mAs, and matrix size of 512 × 512; a 320-channel multidetector CT scanner (Aquilion ONE; Toshiba, Tokyo, Japan) with scan thickness of 0.5 mm, reconstruction interval of 0.5 mm, tube voltage of 100 kV, tube current of 300 mAs, and matrix size of 512 × 512; a 16-channel multidetector CT scanner (Light speed pro; GE Medical Systems, Milwaukee, WI, USA) with scan thickness of 1.25 mm, reconstruction interval of 1.25 mm, tube voltage of 120 kV, tube current of 300 mAs, and matrix size of 512 × 512.
2. Hospital B (from January 2018 to December 2020): All CTA images were acquired using a 256-channel multidetector CT scanner (iCT; Philips Healthcare, Best, The Netherlands) with scan thickness of 0.9 mm, reconstruction interval of 0.45 mm, tube voltage of 120 kV, tube current of 250 mAs, and matrix size of 512 × 512.
3. Hospital C (from January 2018 to December 2020): All CTA images were acquired using a 64-channel multidetector CT scanner (SOMATOM Definition AS+; Siemens Healthcare Sector, Florsheim, Germany) with scan thickness of 0.75 mm, reconstruction interval of 0.6 mm, tube voltage of 120 kV, tube current of 120 mAs, and matrix size of 512 × 512.
4. Hospital D (from January 2017 to October 2019): All CTA images were acquired using a 320-channel multidetector CT scanner (Aquilion ONE; Toshiba, Tokyo, Japan); with scan thickness of 0.5 mm, reconstruction interval of 0.5 mm, tube voltage of 100 kV, tube current of 300 mAs, and matrix size of 512 × 512.
5. Hospital E (from September 2019 to March 2020): All CTA images were acquired using

a 16-channel multidetector CT scanner (uCT510; United imaging, Shanghai, China) with scan thickness of 1 mm, reconstruction interval of 1 mm, tube voltage of 120 kV, tube current of 160 mAs, and matrix size of 1024×1024.

Supplemental Digital Content 2: Definitions of morphological features

Aneurysm size is the longest cross-sectional length of the aneurysm dome. Vessel size was calculated by averaging the cross-sectional diameter (D) of the vessel adjacent to the aneurysm neck and the D 1.5 times from the aneurysm neck. Aneurysm height is the maximum distance from the aneurysm dome to the center of the aneurysm neck. Perpendicular height was the largest perpendicular distance from the aneurysm dome to the aneurysm neck. AR was determined as the ratio of the aneurysm perpendicular height to the average diameter of the aneurysm neck. SR was defined as the ratio of the aneurysm height and the mean vessel size of all vessels surrounding the aneurysm. Aneurysm angle is the angle between the aneurysm height and the aneurysm neck. Flow angle is the angle between the aneurysm height line and the vector of the centerline of the parent artery. Vessel angle is the angle between the aneurysm neck and the vector of the blood flow.

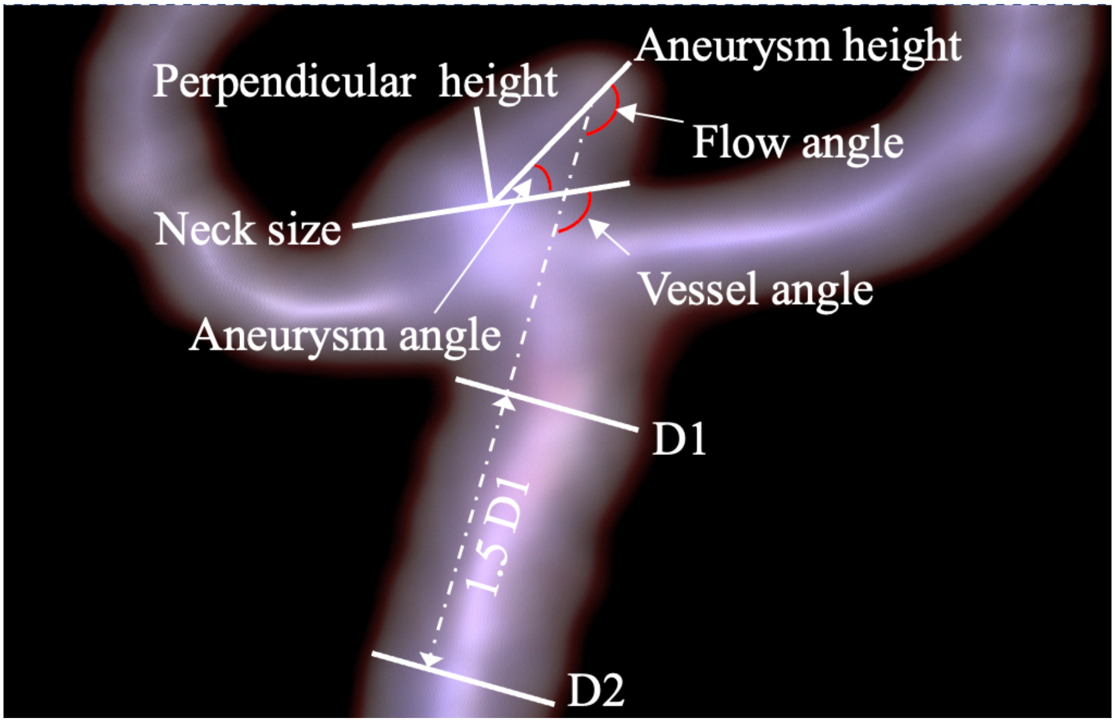

Supplemental Digital Content 3: The possible pathophysiologic meaning of the radiomics features

Feature group	Pathophysiologic meaning of the features
First Order Statistics	First-order statistics describe the distribution of voxel intensities within the image region defined by the mask through commonly used and basic metrics.
Shape-based	In this group of features, we included descriptors of the three-dimensional size and shape of the ROI. These features are independent from the gray level intensity distribution in the ROI and are therefore only calculated on the non-derived image and mask.
GLCM	A Gray Level Co-occurrence Matrix (GLCM) of size Ng Å~ Ng describes the second-order joint probability function of an image region constrained by the mask and is defined as $P(i, j\|\delta, \theta)$. The (i, j)th element of this matrix represents the number of times the combination of levels i and j occur in two pixels in the image, that are separated by a distance of δ pixels along angle θ. The distance δ from the center voxel is defined as the distance according to the infinity norm. For $\delta = 1$, this results in 2 neighbors for each of 13 angles in 3D (26-connectivity) and for $\delta = 2$ a 98-connectivity (49 unique angles).
GLRLM	A Gray Level Run Length Matrix (GLRLM) quantifies gray level runs, which are defined as the length in number of pixels, of consecutive pixels that have the same gray level value. In a gray level run length matrix $P(i, j\|\theta)$, the (i, j)th element describes the number of runs with gray level i and length j occur in the image (ROI) along angle θ.
GLSZM	A Gray Level Size Zone (GLSZM) quantifies gray level zones in an image. A gray level zone is defined as a the number of connected voxels that share the same gray level intensity. A voxel is considered connected if the distance is 1 according to the infinity norm (26-connected region in a 3D, 8-connected region in 2D). In a gray level size zone matrix $P(i, j)$ the (i, j)th element equals the number of zones with gray level i and size j appear in image. Contrary to GLCM and GLRLM, the GLSZM is rotation independent, with only one matrix calculated for all directions in the ROI.
NGTDM	gray value of its neighbors within distance δ. The sum of absolute differences for gray level i is stored in the 2.5. Radiomic Features 59 pyradiomics Documentation, Release 2.1.0 matrix. Let Xgl be a set of segmented voxels and $xgl(jx, jy, jz) \in Xgl$ be the gray level of a voxel at position (jx, jy, jz).
GLDM	A Gray Level Dependence Matrix (GLDM) quantifies gray level dependencies in an image. A gray level dependency is defined as a the number of connected voxels within distance δ that are dependent on the center voxel. A neighboring voxel with gray level j is considered dependent on center voxel with gray level i if $\|i - j\| \leq \alpha$. In a gray level dependence matrix $P(i, j)$ the (i, j)th element describes the number of times a voxel with gray level i with j dependent voxels in its neighborhood appears in image.

GLCM= grey level co-occurrence matrix, GLRLM= grey level run-length matrix, GLSZM= gray level size zone matrix, NGTDM=neighboring gray tone difference matrix, GLDM=gray level dependence matrix.

Supplemental Digital Content 4: The details about radiomics analysis

In regions of interest (ROIs) segmentation procedure, all readers were blinded to the patients' condition. The segmentation process was performed using 3D Slicer software (version 4.10.2; http://www.slicer.org).

In data preprocessing procedure, image resampling and gray-level discretization were used to reduce the variability of radiomics features. Data preprocessing and feature extraction were performed using the Artificial Intelligence Kit software (version 3.0.0.R; ___Healthcare).

Z-score normalization to make the image intensities have the properties of a standard normal distribution by scaling values to a mean of 0 and a standard deviation of 1 using the following formula:

$$z = \frac{\chi - \mu}{\sigma}$$

where μ was the mean value of the images, and σ was the standard deviation of images.

Supplemental Digital Content 5: Detailed information of LASSO and mRMR:

The least absolute shrinkage and selection operator (LASSO) is suitable for feature selection in high-dimensional data. Hence, we used the LASSO method to select the most valuable features. The parameter (λ) is a penalty parameter that varies for each model fitting step. As the value of λ increased, radiomics features with non-zero coefficients decreased. The optimal λ was selected by a 10-fold cross-validation, and the rupture-related radiomics features were subsequently chosen.

The minimum redundancy, maximum relevancy (mRMR) ranking algorithm was performed to select optimal features from the remaining features. Based on mutual information (MI), the mRMR algorithm ranks the input features by maximally distinguish different labels (rupture or non-rupture) while minimizing intra-feature correlation.

Supplemental Digital Content 6: Baseline characteristics of patients in the temporal and external validation dataset

	Temporal validation dataset (N=155)			External validation dataset (N=75)		
Variables	Unruptured (N=72)	Ruptured (N=83)	P value	Unruptured (N=32)	Ruptured (N=43)	P value
Age (y)	65.0 (54.3, 73.0)	56.0 (51.0, 65.0)	.001	67.5 (11.3)	57.9 (11.9)	.001
Female	41.0 (56.9%)	42.0 (50.6%)	.430	18.0 (56.3%)	23.0 (53.5%)	.812
Hypertension [a, b]	36.0 (70.6%)	39.0 (57.4%)	.139	24.0 (75.0%)	22.0 (57.9%)	.113
Smoking [c, d]	6.0 (11.8%)	9.0 (13.2%)	.811	8.0 (28.6%)	14.0 (43.8%)	.224
Location [e]			.093			.456
M1	26.0 (36.1%)	27.0 (32.5%)		6.0 (19.4%)	7.0 (16.3%)	
Mbif	36.0 (50.0%)	52.0 (62.7%)		24.0 (77.4%)	36.0 (83.7%)	
Mdist	10.0 (13.9%)	4.0 (4.8%)		1.0 (3.2%)	0 (0%)	
Side			.310			.217
Right	44.0 (61.1%)	44.0 (53.0%)		18.0 (56.3%)	18.0 (41.9%)	
Left	28.0 (38.9%)	39.0 (47.0%)		14.0 (43.8%)	25.0 (58.1%)	
Multiplicity	23.0 (31.9%)	7.0 (8.4%)	< .001	8.0 (25.0%)	1.0 (2.3%)	0.009
Vessel size (mm)	2.4 (0.5)	2.3 (0.4)	.176	2.3 (0.5)	2.0 (0.5)	.023
Aneurysm size (mm)	5.0 (3.9, 7.1)	7.1 (5.1, 9.2)	< .001	4.6 (3.9, 6.8)	5.9 (4.4, 8.0)	.085
Neck size (mm)	4.3 (3.3, 5.6)	4.1 (3.4, 5.5)	.889	3.7 (3.3, 4.6)	3.9 (3.1, 4.9)	.962
Aspect ratio	0.7 (0.4, 0.9)	0.9 (0.7, 1.2)	< .001	0.7 (0.6, 1.0)	0.9 (0.7, 1.2)	.089
Size ratio	1.3 (0.9, 2,2)	2.1 (1.6, 3.2)	< .001	1.5 (1.1, 2.4)	2.7 (1.6, 3.7)	.003
Aneurysm height (mm)	3.4 (2.3, 4.7)	4.8 (3.6, 7.1)	< .001	3.3 (2.8, 5.5)	5.0 (3.3, 6.3)	.010
Perpendicular height (mm)	2.9 (2.0, 4.2)	4.1 (3.0, 5.3)	< .001	3.0 (2.3, 4.9)	3.9 (2.9, 5.5)	.068
Aneurysm angle (°)	68.3 (53.5, 88.9)	65.9 (54.2, 77.6)	.241	61.7 (51.7, 90.0)	69.2 (53.3, 76.6)	.723
Vessel angle (°)	58.5 (29.1, 74.2)	57.3 (40.8, 74.7)	.589	57.2 (23.8, 79.2)	54.6 (27.5, 75.4)	.822
Flow angle (°)	139.1 (114.9, 158.8)	148.1 (131.5, 168.7)	.017	128.0 (102.8, 151.7)	135.7 (113.8, 161.9)	.400
Daughter dome	8.0 (11.1%)	18.0 (21.7%)	.079	3.0 (9.4%)	10.0 (23.3%)	.116
Irregular shape	15.0 (20.8%)	40.0 (48.2%)	< .001	13.0 (40.6%)	24.0 (55.8%)	.193

[a] 36/155 (23.23%) missing values for temporal validation dataset.

[b] 5/75 (6.67%) missing values for external validation dataset.

[c] 36/155 (23.23%) missing values for temporal validation dataset.

[d] 15/75 (20.0%) missing values for external validation dataset.

[e] 1/75 (1.33%) missing values for external validation dataset.

Supplemental Digital Content 7: Radiomics signature calculation formula

Rad score= 0.760194428×wavelet-HHL_glcm_ClusterTendency

+0.214848210×wavelet-LLH_firstorder_Variance

-0.050853945×wavelet-LHL_gldm_DependenceNonUniformityNormalized

-0.092363871×wavelet-HHL_firstorder_Kurtosis

-0.045566829×wavelet-HLL_ngtdm_Contrast

-0.084773992×wavelet-LLH_glcm_ClusterShade

-0.006274138 wavelet-LHH_glcm_ClusterShade
